# (1*R*,4*R*,5*R*)-1,3,4-Triphenyl-7-[(*R*)-1-phenyl­ethyl]-2-oxa-3,7-diaza­spiro­[4.5]decan-10-one

**DOI:** 10.1107/S1600536807067256

**Published:** 2007-12-21

**Authors:** A. Malathy, R. Suresh Kumar, S. Perumal, J. Suresh, Nilantha Lakshman

**Affiliations:** a Department of Physics, Madurai Kamaraj University, Madurai 625 021, India; b School of Chemistry, Madurai Kamaraj University, Madurai 625 021, India; c Department of Physics, Madura College, Madurai 625 011, India; d Department of Food science and Technology, Faculty of Agriculture, University of Ruhuna, Mapalana, Kamburupitiya 81100, Sri Lanka

## Abstract

In the title compound, C_33_H_32_N_2_O_2_, the polysubstituted piperidine ring adopts a chair conformation. The isoxazolidine ring is in an envelope conformation. In the crystal structure, intra- and inter­molecular C—H⋯π inter­actions involving the phenyl rings are observed.

## Related literature

For related literature, see: Ali Dondas *et al.* (2001 ▶); Alibés *et al.* (2003 ▶); Blanarikova-Hlobilova *et al.* (2003 ▶); Carda *et al.* (2000 ▶); Carruthers (1990 ▶); Herrera *et al.* (2001 ▶); Huisgen (1963 ▶); Ishar *et al.* (2000 ▶). For ring puckering parameters, see: Cremer & Pople (1975 ▶).
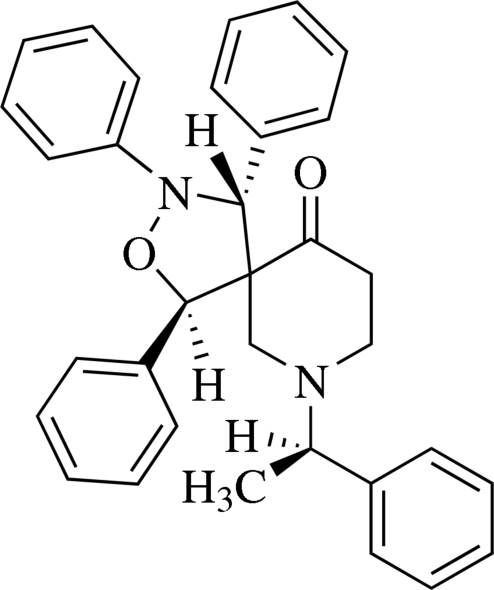


## Experimental

### 

#### Crystal data

C_33_H_32_N_2_O_2_
*M* *_r_* = 488.61
Orthorhombic, 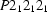

*a* = 10.589 (5) Å
*b* = 14.582 (7) Å
*c* = 17.443 (8) Å
*V* = 2693 (2) Å^3^
*Z* = 4
Mo *K*α radiation
μ = 0.08 mm^−1^
*T* = 273 (2) K
0.20 × 0.16 × 0.12 mm

#### Data collection

Nonius MACH-3 diffractometer
Absorption correction: ψ scan (North *et al.*, 1968 ▶) *T*_min_ = 0.986, *T*_max_ = 0.991
13617 measured reflections
2701 independent reflections
1899 reflections with *I* > 2σ(*I*)
*R*_int_ = 0.074
2 standard reflections frequency: 60 min intensity decay: none

#### Refinement

*R*[*F* ^2^ > 2σ(*F* ^2^)] = 0.065
*wR*(*F* ^2^) = 0.118
*S* = 1.09
2701 reflections
335 parameters
H-atom parameters constrained
Δρ_max_ = 0.16 e Å^−3^
Δρ_min_ = −0.14 e Å^−3^

### 

Data collection: *CAD-4 EXPRESS* (Enraf–Nonius, 1994 ▶); cell refinement: *CAD-4 EXPRESS*; data reduction: *XCAD4* (Harms & Wocadlo, 1996 ▶); program(s) used to solve structure: *SHELXS97* (Sheldrick, 1997 ▶); program(s) used to refine structure: *SHELXL97* (Sheldrick, 1997 ▶); molecular graphics: *PLATON* (Spek, 2003 ▶); software used to prepare material for publication: *SHELXL97*.

## Supplementary Material

Crystal structure: contains datablocks global, I. DOI: 10.1107/S1600536807067256/ci2540sup1.cif

Structure factors: contains datablocks I. DOI: 10.1107/S1600536807067256/ci2540Isup2.hkl

Additional supplementary materials:  crystallographic information; 3D view; checkCIF report

## Figures and Tables

**Table 1:** Hydrogen-bond geometry (Å, °) *Cg*1, *Cg*2 and *Cg*3 are the centroids of the phenyl rings C71–C76, C91–C96 and C81–C86, respectively.

*D*—H⋯*A*	*D*—H	H⋯*A*	*D*⋯*A*	*D*—H⋯*A*
C8—H8⋯O1	0.98	2.35	2.775 (5)	106
C26—H26⋯O2	0.93	2.29	2.623 (5)	101
C82—H82⋯O2	0.93	2.43	2.757 (5)	101
C3—H3*A*⋯*Cg*1	0.97	2.90	3.659 (5)	136
C2—H2*A*⋯*Cg*2^i^	0.97	2.93	3.707 (5)	138
C74—H74⋯*Cg*3^ii^	0.93	2.96	3.722 (6)	141

Symmetry codes: (i) 
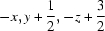
; (ii) 
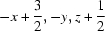
.

**Fig. 1.**
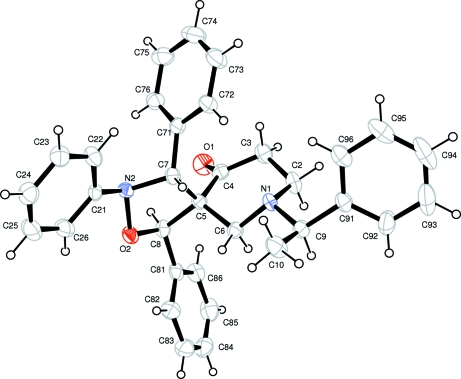
The molecular structure of the title compound, showing 30% probability displacement ellipsoids and the atom-numbering scheme.

## supplementary crystallographic information

### Comment

1,3-Dipolar cycloaddition is a versatile reaction for the construction of five-membered ring heterocycles of biological importance (Huisgen, 1963). Among the 1,3-dipoles, nitrones have been subjected to numerous 1,3-dipolar cycloadditions, ascribable to their stability and ease of generation (Blanarikova-Hlobilova *et al.*, 2003; Herrera *et al.*, 2001). The 1,3-dipolar cycloaddition of nitrones to alkenes afford isoxazolidines with generation of as many as three new contiguous stereocenters in a single step (Ishar *et al.*, 2000; Carda *et al.*, 2000; Ali Dondas *et al.*, 2001; Alibés *et al.*, 2003). These isoxazolidines can be further elaborated into polyfunctional cyclic or acyclic bioactive compounds with complete control of relative stereochemistry (Carruthers, 1990).

The molecular structure of (I) is shown in Fig.1. The five-membered isoxazolidine ring has an envelope conformation, as indicated by the Cremer & Pople (1975) puckering parameters Q = 0.454 (3) Å and φ = 3.3 (5)°. The piperidine ring adopts a chair conformation. The dihedral angle between the C21–C26 and C71–C76 phenyl rings is 77.7 (1)°. The C21–C26, C71–C76 and C81–C86 phenyl rings form dihedral angles of 35.8 (2)°, 77.5 (1)° and 72.3 (2)°, respectively, with the N2/C7/C5/C8 plane.

Weak intramolecular C—H···O and C—H···π interactions are observed in the molecular structure. The packing of molecules is governed by weak C—H···π interactions (Table 1) and van der Walls interactions. In the Table 1, *Cg*1, *Cg*2 and *Cg*3 denote the centroids of the C71–C76, C91–C96 and C81–C86 phenyl rings.

### Experimental

A mixture of [(*R*)-1-phenylethyl]-3-[(*E*)-phenylmethylidene]tetrahydro-4(1*H*)- pyridinone (0.300 g, 1 mmol) and nitrone (0.244 g, 1.2 mmol) in toluene (25 ml) was refluxed for 10 h. The progress of the reaction was monitored by thin-layer chromatography (TLC) and after completion of the reaction, the solvent was evaporated *in vacuo*. The residue was then subjected to flash column chromatography on silica gel using petroleum ether-ethyl acetate (10:1) as eluent to obtain crystals of the title compound in 8% yield (0.040 g) along with two other products in semi-solid form.

### Refinement

H atoms were placed at calculated positions and allowed to ride on their carrier atoms with C—H = 0.93–0.98 Å, and *U*_iso_ = 1.2*U*_eq_(C) for CH_2_ and CH groups, and 1.5*U*_eq_ for CH_3_ groups. In the absence of significant anomalous scattering, the absolute configuration could not be reliably determined and Friedel pairs were merged.

### Crystal data

**Table:**

C_33_H_32_N_2_O_2_	*F*_000_ = 1040
*M**_r_* = 488.61	*D*_x_ = 1.205 Mg m^−^^3^
Orthorhombic, *P*2_1_2_1_2_1_	Mo *K*α radiation λ = 0.71073 Å
Hall symbol: P 2ac 2ab	Cell parameters from 25 reflections
*a* = 10.589 (5) Å	θ = 2–25º
*b* = 14.582 (7) Å	µ = 0.08 mm^−^^1^
*c* = 17.443 (8) Å	*T* = 273 (2) K
*V* = 2693 (2) Å^3^	Needle, colourless
*Z* = 4	0.20 × 0.16 × 0.12 mm

### Data collection

**Table:**

Nonius MACH-3 diffractometer	*R*_int_ = 0.074
Radiation source: fine-focus sealed tube	θ_max_ = 25.0º
Monochromator: graphite	θ_min_ = 1.8º
*T* = 273(2) K	*h* = −12→11
ω/2θ scans	*k* = −17→16
Absorption correction: ψ scan(North *et al.*, 1968)	*l* = −20→20
*T*_min_ = 0.986, *T*_max_ = 0.991	2 standard reflections
13617 measured reflections	every 60 min
2701 independent reflections	intensity decay: none
1899 reflections with *I* > 2σ(*I*)	

### Refinement

**Table:**

Refinement on *F*^2^	Secondary atom site location: difference Fourier map
Least-squares matrix: full	Hydrogen site location: inferred from neighbouring sites
*R*[*F*^2^ > 2σ(*F*^2^)] = 0.065	H-atom parameters constrained
*wR*(*F*^2^) = 0.118	*w* = 1/[σ^2^(*F*_o_^2^) + (0.0519*P*)^2^] where *P* = (*F*_o_^2^ + 2*F*_c_^2^)/3
*S* = 1.09	(Δ/σ)_max_ < 0.001
2701 reflections	Δρ_max_ = 0.16 e Å^−^^3^
335 parameters	Δρ_min_ = −0.14 e Å^−^^3^
Primary atom site location: structure-invariant direct methods	Extinction correction: none

### Special details

**Table:**

Geometry. All e.s.d.'s (except the e.s.d. in the dihedral angle between two l.s. planes) are estimated using the full covariance matrix. The cell e.s.d.'s are taken into account individually in the estimation of e.s.d.'s in distances, angles and torsion angles; correlations between e.s.d.'s in cell parameters are only used when they are defined by crystal symmetry. An approximate (isotropic) treatment of cell e.s.d.'s is used for estimating e.s.d.'s involving l.s. planes.
Refinement. Refinement of *F*^2^ against ALL reflections. The weighted *R*-factor *wR* and goodness of fit *S* are based on *F*^2^, conventional *R*-factors *R* are based on *F*, with *F* set to zero for negative *F*^2^. The threshold expression of *F*^2^ > σ(*F*^2^) is used only for calculating *R*-factors(gt) *etc*. and is not relevant to the choice of reflections for refinement. *R*-factors based on *F*^2^ are statistically about twice as large as those based on *F*, and *R*- factors based on ALL data will be even larger.

### Fractional atomic coordinates and isotropic or equivalent isotropic displacement parameters (Å^2^)

**Table:**

	*x*	*y*	*z*	*U*_iso_*/*U*_eq_	
C2	0.1883 (4)	0.3386 (3)	0.0817 (2)	0.0554 (11)	
H2A	0.1260	0.2938	0.0978	0.066*	
H2B	0.1896	0.3399	0.0261	0.066*	
C3	0.1521 (4)	0.4324 (3)	0.1120 (2)	0.0544 (11)	
H3A	0.2006	0.4784	0.0847	0.065*	
H3B	0.0636	0.4430	0.1007	0.065*	
C4	0.1724 (4)	0.4453 (3)	0.1961 (2)	0.0443 (10)	
C5	0.2865 (3)	0.3966 (2)	0.2314 (2)	0.0390 (9)	
C6	0.3004 (4)	0.3021 (2)	0.1937 (2)	0.0428 (10)	
H6A	0.3743	0.2713	0.2142	0.051*	
H6B	0.2269	0.2649	0.2054	0.051*	
C7	0.4141 (4)	0.4506 (2)	0.22225 (19)	0.0406 (9)	
H7	0.4773	0.4089	0.2006	0.049*	
C8	0.2734 (4)	0.3908 (3)	0.3199 (2)	0.0438 (10)	
H8	0.2265	0.4444	0.3382	0.053*	
C9	0.3577 (4)	0.2247 (3)	0.0750 (2)	0.0481 (11)	
H9	0.3031	0.1748	0.0928	0.058*	
C10	0.4920 (4)	0.2034 (3)	0.0992 (2)	0.0643 (13)	
H10A	0.4945	0.1932	0.1535	0.096*	
H10B	0.5457	0.2542	0.0864	0.096*	
H10C	0.5208	0.1494	0.0730	0.096*	
C21	0.5823 (4)	0.4778 (2)	0.31879 (19)	0.0383 (9)	
C22	0.6625 (4)	0.5250 (3)	0.2706 (2)	0.0528 (10)	
H22	0.6305	0.5506	0.2258	0.063*	
C23	0.7890 (4)	0.5351 (3)	0.2874 (2)	0.0562 (11)	
H23	0.8411	0.5680	0.2544	0.067*	
C24	0.8378 (4)	0.4969 (3)	0.3523 (3)	0.0617 (12)	
H24	0.9233	0.5027	0.3635	0.074*	
C25	0.7593 (5)	0.4500 (3)	0.4008 (3)	0.0723 (14)	
H25	0.7921	0.4239	0.4452	0.087*	
C26	0.6322 (4)	0.4408 (3)	0.3848 (2)	0.0590 (12)	
H26	0.5800	0.4096	0.4188	0.071*	
C71	0.4083 (4)	0.5349 (3)	0.1730 (2)	0.0429 (9)	
C72	0.4473 (4)	0.5300 (3)	0.0971 (2)	0.0573 (12)	
H72	0.4842	0.4764	0.0788	0.069*	
C73	0.4319 (5)	0.6035 (4)	0.0490 (3)	0.0742 (14)	
H73	0.4565	0.5987	−0.0021	0.089*	
C74	0.3810 (5)	0.6836 (4)	0.0748 (3)	0.0781 (16)	
H74	0.3696	0.7329	0.0417	0.094*	
C75	0.3467 (4)	0.6905 (3)	0.1509 (3)	0.0673 (13)	
H75	0.3150	0.7456	0.1696	0.081*	
C76	0.3589 (4)	0.6165 (3)	0.1994 (2)	0.0523 (11)	
H76	0.3336	0.6216	0.2502	0.063*	
C81	0.2124 (4)	0.3053 (3)	0.3508 (2)	0.0453 (10)	
C82	0.2819 (4)	0.2315 (3)	0.3763 (2)	0.0559 (12)	
H82	0.3696	0.2350	0.3767	0.067*	
C83	0.2234 (5)	0.1530 (3)	0.4014 (3)	0.0680 (14)	
H83	0.2718	0.1037	0.4182	0.082*	
C84	0.0949 (6)	0.1466 (4)	0.4019 (2)	0.0737 (15)	
H84	0.0559	0.0933	0.4191	0.088*	
C85	0.0232 (5)	0.2193 (4)	0.3767 (3)	0.0758 (15)	
H85	−0.0644	0.2153	0.3766	0.091*	
C86	0.0824 (4)	0.2987 (3)	0.3515 (2)	0.0571 (12)	
H86	0.0339	0.3481	0.3349	0.068*	
C91	0.3510 (4)	0.2279 (3)	−0.0116 (2)	0.0496 (11)	
C92	0.3106 (5)	0.1525 (3)	−0.0517 (3)	0.0711 (14)	
H92	0.2824	0.1011	−0.0253	0.085*	
C93	0.3112 (6)	0.1519 (5)	−0.1306 (3)	0.099 (2)	
H93	0.2846	0.0998	−0.1568	0.119*	
C94	0.3506 (6)	0.2269 (6)	−0.1706 (3)	0.103 (2)	
H94	0.3513	0.2259	−0.2239	0.124*	
C95	0.3891 (5)	0.3036 (5)	−0.1320 (3)	0.0862 (17)	
H95	0.4133	0.3559	−0.1588	0.103*	
C96	0.3919 (4)	0.3029 (4)	−0.0530 (2)	0.0661 (13)	
H96	0.4221	0.3542	−0.0270	0.079*	
N1	0.3132 (3)	0.3116 (2)	0.11050 (15)	0.0403 (8)	
N2	0.4506 (3)	0.4721 (2)	0.30247 (16)	0.0423 (8)	
O1	0.1026 (3)	0.4923 (2)	0.23424 (16)	0.0660 (8)	
O2	0.4005 (2)	0.39618 (18)	0.34690 (13)	0.0483 (7)	

### Atomic displacement parameters (Å^2^)

**Table:**

	*U*^11^	*U*^22^	*U*^33^	*U*^12^	*U*^13^	*U*^23^
C2	0.045 (3)	0.080 (3)	0.041 (2)	0.002 (3)	−0.006 (2)	−0.009 (2)
C3	0.037 (2)	0.071 (3)	0.055 (3)	0.007 (2)	−0.009 (2)	0.000 (2)
C4	0.039 (2)	0.043 (2)	0.051 (3)	−0.004 (2)	0.004 (2)	0.002 (2)
C5	0.041 (2)	0.041 (2)	0.035 (2)	−0.001 (2)	0.0020 (17)	0.0005 (18)
C6	0.044 (2)	0.042 (2)	0.042 (2)	0.001 (2)	0.0011 (19)	−0.0026 (19)
C7	0.040 (2)	0.046 (2)	0.036 (2)	0.000 (2)	0.0019 (18)	−0.0049 (19)
C8	0.042 (2)	0.051 (2)	0.039 (2)	−0.002 (2)	0.0009 (18)	−0.007 (2)
C9	0.059 (3)	0.049 (2)	0.036 (2)	−0.006 (2)	0.007 (2)	−0.0012 (19)
C10	0.074 (3)	0.074 (3)	0.044 (3)	0.025 (3)	0.009 (2)	−0.002 (2)
C21	0.045 (2)	0.037 (2)	0.033 (2)	0.000 (2)	−0.0034 (19)	−0.0061 (18)
C22	0.050 (3)	0.070 (3)	0.038 (2)	−0.001 (2)	0.000 (2)	0.003 (2)
C23	0.053 (3)	0.064 (3)	0.052 (3)	−0.006 (2)	0.005 (2)	−0.001 (2)
C24	0.046 (3)	0.068 (3)	0.071 (3)	−0.004 (3)	−0.007 (2)	−0.002 (3)
C25	0.066 (3)	0.083 (3)	0.068 (3)	−0.002 (3)	−0.025 (3)	0.015 (3)
C26	0.060 (3)	0.065 (3)	0.052 (3)	−0.011 (2)	−0.005 (2)	0.015 (2)
C71	0.041 (2)	0.046 (2)	0.042 (2)	−0.007 (2)	−0.0045 (19)	0.002 (2)
C72	0.069 (3)	0.062 (3)	0.041 (3)	−0.009 (2)	−0.001 (2)	0.007 (2)
C73	0.085 (4)	0.090 (4)	0.048 (3)	−0.012 (3)	−0.003 (3)	0.017 (3)
C74	0.072 (4)	0.082 (4)	0.080 (4)	−0.012 (3)	−0.011 (3)	0.040 (3)
C75	0.060 (3)	0.059 (3)	0.084 (4)	0.001 (3)	−0.005 (3)	0.016 (3)
C76	0.050 (3)	0.056 (3)	0.050 (3)	−0.007 (2)	0.001 (2)	0.006 (2)
C81	0.051 (3)	0.056 (3)	0.029 (2)	−0.004 (2)	0.0061 (19)	−0.005 (2)
C82	0.055 (3)	0.063 (3)	0.050 (3)	−0.002 (3)	0.009 (2)	0.005 (2)
C83	0.087 (4)	0.060 (3)	0.056 (3)	−0.003 (3)	0.005 (3)	0.006 (3)
C84	0.095 (4)	0.071 (3)	0.055 (3)	−0.034 (4)	0.010 (3)	−0.002 (3)
C85	0.058 (3)	0.104 (4)	0.066 (3)	−0.024 (3)	0.002 (3)	−0.005 (3)
C86	0.049 (3)	0.071 (3)	0.051 (3)	−0.004 (3)	0.003 (2)	0.001 (2)
C91	0.051 (3)	0.061 (3)	0.037 (2)	0.008 (2)	0.002 (2)	−0.005 (2)
C92	0.084 (4)	0.068 (3)	0.061 (3)	0.006 (3)	−0.009 (3)	−0.024 (3)
C93	0.105 (5)	0.125 (5)	0.068 (4)	0.033 (5)	−0.027 (4)	−0.047 (4)
C94	0.105 (5)	0.165 (7)	0.040 (3)	0.061 (5)	−0.010 (3)	−0.012 (4)
C95	0.086 (4)	0.122 (5)	0.050 (4)	0.025 (4)	0.010 (3)	0.024 (3)
C96	0.070 (3)	0.084 (3)	0.044 (3)	0.008 (3)	0.004 (2)	0.004 (3)
N1	0.0415 (19)	0.0506 (18)	0.0287 (18)	0.0008 (17)	−0.0027 (14)	−0.0007 (15)
N2	0.050 (2)	0.0451 (19)	0.0324 (18)	−0.0084 (16)	0.0004 (14)	0.0052 (16)
O1	0.0558 (18)	0.073 (2)	0.0693 (19)	0.0207 (17)	0.0077 (16)	−0.0070 (17)
O2	0.0513 (17)	0.0588 (17)	0.0346 (14)	−0.0115 (15)	−0.0023 (13)	0.0056 (14)

### Geometric parameters (Å, °)

**Table:**

C2—N1	1.468 (5)	C25—C26	1.382 (6)
C2—C3	1.515 (5)	C25—H25	0.93
C2—H2A	0.97	C26—H26	0.93
C2—H2B	0.97	C71—C76	1.379 (5)
C3—C4	1.495 (5)	C71—C72	1.388 (5)
C3—H3A	0.97	C72—C73	1.372 (6)
C3—H3B	0.97	C72—H72	0.93
C4—O1	1.208 (4)	C73—C74	1.363 (7)
C4—C5	1.530 (5)	C73—H73	0.93
C5—C6	1.534 (5)	C74—C75	1.379 (6)
C5—C8	1.552 (5)	C74—H74	0.93
C5—C7	1.572 (5)	C75—C76	1.376 (5)
C6—N1	1.465 (4)	C75—H75	0.93
C6—H6A	0.97	C76—H76	0.93
C6—H6B	0.97	C81—C82	1.378 (5)
C7—N2	1.485 (4)	C81—C86	1.380 (5)
C7—C71	1.502 (5)	C82—C83	1.374 (6)
C7—H7	0.98	C82—H82	0.93
C8—O2	1.428 (4)	C83—C84	1.364 (7)
C8—C81	1.503 (5)	C83—H83	0.93
C8—H8	0.98	C84—C85	1.375 (7)
C9—N1	1.488 (5)	C84—H84	0.93
C9—C91	1.512 (5)	C85—C86	1.388 (6)
C9—C10	1.516 (6)	C85—H85	0.93
C9—H9	0.98	C86—H86	0.93
C10—H10A	0.96	C91—C92	1.372 (5)
C10—H10B	0.96	C91—C96	1.381 (6)
C10—H10C	0.96	C92—C93	1.375 (6)
C21—C26	1.376 (5)	C92—H92	0.93
C21—C22	1.379 (5)	C93—C94	1.363 (8)
C21—N2	1.425 (4)	C93—H93	0.93
C22—C23	1.380 (6)	C94—C95	1.369 (8)
C22—H22	0.93	C94—H94	0.93
C23—C24	1.365 (5)	C95—C96	1.378 (6)
C23—H23	0.93	C95—H95	0.93
C24—C25	1.369 (6)	C96—H96	0.93
C24—H24	0.93	N2—O2	1.452 (4)
			
N1—C2—C3	110.5 (3)	C26—C25—H25	119.5
N1—C2—H2A	109.5	C21—C26—C25	120.4 (4)
C3—C2—H2A	109.5	C21—C26—H26	119.8
N1—C2—H2B	109.5	C25—C26—H26	119.8
C3—C2—H2B	109.5	C76—C71—C72	118.4 (4)
H2A—C2—H2B	108.1	C76—C71—C7	122.1 (3)
C4—C3—C2	114.8 (3)	C72—C71—C7	119.4 (4)
C4—C3—H3A	108.6	C73—C72—C71	120.5 (4)
C2—C3—H3A	108.6	C73—C72—H72	119.8
C4—C3—H3B	108.6	C71—C72—H72	119.8
C2—C3—H3B	108.6	C74—C73—C72	120.9 (4)
H3A—C3—H3B	107.5	C74—C73—H73	119.5
O1—C4—C3	121.5 (4)	C72—C73—H73	119.5
O1—C4—C5	121.7 (4)	C73—C74—C75	119.0 (4)
C3—C4—C5	116.7 (3)	C73—C74—H74	120.5
C4—C5—C6	108.7 (3)	C75—C74—H74	120.5
C4—C5—C8	110.8 (3)	C76—C75—C74	120.6 (5)
C6—C5—C8	112.7 (3)	C76—C75—H75	119.7
C4—C5—C7	113.9 (3)	C74—C75—H75	119.7
C6—C5—C7	108.9 (3)	C75—C76—C71	120.5 (4)
C8—C5—C7	101.9 (3)	C75—C76—H76	119.8
N1—C6—C5	110.4 (3)	C71—C76—H76	119.8
N1—C6—H6A	109.6	C82—C81—C86	118.4 (4)
C5—C6—H6A	109.6	C82—C81—C8	122.2 (4)
N1—C6—H6B	109.6	C86—C81—C8	119.3 (4)
C5—C6—H6B	109.6	C83—C82—C81	120.8 (4)
H6A—C6—H6B	108.1	C83—C82—H82	119.6
N2—C7—C71	112.1 (3)	C81—C82—H82	119.6
N2—C7—C5	103.5 (3)	C84—C83—C82	120.6 (5)
C71—C7—C5	115.7 (3)	C84—C83—H83	119.7
N2—C7—H7	108.4	C82—C83—H83	119.7
C71—C7—H7	108.4	C83—C84—C85	119.7 (5)
C5—C7—H7	108.4	C83—C84—H84	120.1
O2—C8—C81	109.4 (3)	C85—C84—H84	120.1
O2—C8—C5	103.9 (3)	C84—C85—C86	119.7 (5)
C81—C8—C5	116.1 (3)	C84—C85—H85	120.2
O2—C8—H8	109.0	C86—C85—H85	120.2
C81—C8—H8	109.0	C81—C86—C85	120.7 (5)
C5—C8—H8	109.0	C81—C86—H86	119.6
N1—C9—C91	112.0 (3)	C85—C86—H86	119.6
N1—C9—C10	110.8 (3)	C92—C91—C96	117.7 (4)
C91—C9—C10	109.2 (3)	C92—C91—C9	120.0 (4)
N1—C9—H9	108.3	C96—C91—C9	122.2 (4)
C91—C9—H9	108.3	C91—C92—C93	121.0 (5)
C10—C9—H9	108.3	C91—C92—H92	119.5
C9—C10—H10A	109.5	C93—C92—H92	119.5
C9—C10—H10B	109.5	C94—C93—C92	120.6 (6)
H10A—C10—H10B	109.5	C94—C93—H93	119.7
C9—C10—H10C	109.5	C92—C93—H93	119.7
H10A—C10—H10C	109.5	C93—C94—C95	119.6 (5)
H10B—C10—H10C	109.5	C93—C94—H94	120.2
C26—C21—C22	117.9 (4)	C95—C94—H94	120.2
C26—C21—N2	121.3 (4)	C94—C95—C96	119.6 (6)
C22—C21—N2	120.6 (3)	C94—C95—H95	120.2
C21—C22—C23	121.5 (4)	C96—C95—H95	120.2
C21—C22—H22	119.2	C95—C96—C91	121.5 (5)
C23—C22—H22	119.2	C95—C96—H96	119.3
C24—C23—C22	120.0 (4)	C91—C96—H96	119.3
C24—C23—H23	120.0	C6—N1—C2	106.3 (3)
C22—C23—H23	120.0	C6—N1—C9	111.2 (3)
C23—C24—C25	119.2 (4)	C2—N1—C9	111.8 (3)
C23—C24—H24	120.4	C21—N2—O2	107.2 (3)
C25—C24—H24	120.4	C21—N2—C7	117.1 (3)
C24—C25—C26	121.0 (4)	O2—N2—C7	104.3 (2)
C24—C25—H25	119.5	C8—O2—N2	102.2 (3)
			
N1—C2—C3—C4	−47.1 (5)	O2—C8—C81—C82	−21.0 (5)
C2—C3—C4—O1	−145.8 (4)	C5—C8—C81—C82	96.2 (4)
C2—C3—C4—C5	34.6 (5)	O2—C8—C81—C86	161.1 (3)
O1—C4—C5—C6	142.2 (3)	C5—C8—C81—C86	−81.7 (5)
C3—C4—C5—C6	−38.3 (4)	C86—C81—C82—C83	0.5 (6)
O1—C4—C5—C8	17.9 (5)	C8—C81—C82—C83	−177.4 (4)
C3—C4—C5—C8	−162.6 (3)	C81—C82—C83—C84	−0.4 (7)
O1—C4—C5—C7	−96.3 (4)	C82—C83—C84—C85	0.3 (7)
C3—C4—C5—C7	83.3 (4)	C83—C84—C85—C86	−0.4 (7)
C4—C5—C6—N1	57.2 (4)	C82—C81—C86—C85	−0.6 (6)
C8—C5—C6—N1	−179.7 (3)	C8—C81—C86—C85	177.4 (3)
C7—C5—C6—N1	−67.4 (4)	C84—C85—C86—C81	0.5 (7)
C4—C5—C7—N2	116.1 (3)	N1—C9—C91—C92	−139.4 (4)
C6—C5—C7—N2	−122.4 (3)	C10—C9—C91—C92	97.5 (5)
C8—C5—C7—N2	−3.2 (3)	N1—C9—C91—C96	44.8 (5)
C4—C5—C7—C71	−6.9 (4)	C10—C9—C91—C96	−78.4 (5)
C6—C5—C7—C71	114.5 (3)	C96—C91—C92—C93	0.2 (7)
C8—C5—C7—C71	−126.3 (3)	C9—C91—C92—C93	−175.9 (4)
C4—C5—C8—O2	−147.5 (3)	C91—C92—C93—C94	−0.9 (9)
C6—C5—C8—O2	90.5 (4)	C92—C93—C94—C95	−0.4 (9)
C7—C5—C8—O2	−26.0 (4)	C93—C94—C95—C96	2.3 (9)
C4—C5—C8—C81	92.2 (4)	C94—C95—C96—C91	−3.1 (8)
C6—C5—C8—C81	−29.7 (5)	C92—C91—C96—C95	1.8 (7)
C7—C5—C8—C81	−146.2 (3)	C9—C91—C96—C95	177.8 (4)
C26—C21—C22—C23	0.2 (6)	C5—C6—N1—C2	−71.9 (4)
N2—C21—C22—C23	176.7 (4)	C5—C6—N1—C9	166.2 (3)
C21—C22—C23—C24	0.9 (6)	C3—C2—N1—C6	64.9 (4)
C22—C23—C24—C25	−1.0 (6)	C3—C2—N1—C9	−173.7 (3)
C23—C24—C25—C26	0.1 (7)	C91—C9—N1—C6	170.6 (3)
C22—C21—C26—C25	−1.1 (6)	C10—C9—N1—C6	−67.2 (4)
N2—C21—C26—C25	−177.6 (4)	C91—C9—N1—C2	52.0 (4)
C24—C25—C26—C21	1.0 (7)	C10—C9—N1—C2	174.1 (3)
N2—C7—C71—C76	−40.4 (5)	C26—C21—N2—O2	−21.8 (4)
C5—C7—C71—C76	78.0 (4)	C22—C21—N2—O2	161.8 (3)
N2—C7—C71—C72	143.3 (4)	C26—C21—N2—C7	−138.4 (3)
C5—C7—C71—C72	−98.4 (4)	C22—C21—N2—C7	45.2 (5)
C76—C71—C72—C73	−2.7 (6)	C71—C7—N2—C21	−85.5 (4)
C7—C71—C72—C73	173.8 (4)	C5—C7—N2—C21	149.1 (3)
C71—C72—C73—C74	1.6 (7)	C71—C7—N2—O2	156.3 (3)
C72—C73—C74—C75	1.0 (8)	C5—C7—N2—O2	31.0 (3)
C73—C74—C75—C76	−2.5 (7)	C81—C8—O2—N2	170.9 (3)
C74—C75—C76—C71	1.4 (7)	C5—C8—O2—N2	46.2 (3)
C72—C71—C76—C75	1.2 (6)	C21—N2—O2—C8	−173.9 (3)
C7—C71—C76—C75	−175.2 (4)	C7—N2—O2—C8	−49.1 (3)

### Hydrogen-bond geometry (Å, °)

**Table:**

*D*—H···*A*	*D*—H	H···*A*	*D*···*A*	*D*—H···*A*
C8—H8···O1	0.98	2.35	2.775 (5)	106
C26—H26···O2	0.93	2.29	2.623 (5)	101
C82—H82···O2	0.93	2.43	2.757 (5)	101
C3—H3A···Cg1	0.97	2.90	3.659 (5)	136
C2—H2A···Cg2^i^	0.97	2.93	3.707 (5)	138
C74—H74···Cg3^ii^	0.93	2.96	3.722 (6)	141

Symmetry codes: (i) −*x*, *y*+1/2, −*z*+3/2; (ii) −*x*+3/2, −*y*, *z*+1/2.
